# The World’s Workers

**Published:** 1873-03

**Authors:** 


HALL'S

JOURNAL OF HEALTH.

VOL. XX.—MARCH, 1873.—NO. III.

### THE WORLD'S WORKERS.

A CLERGYMAN of sixty, educated, and with a high sense of honor, gave up his ministrations and went out to day's work, to support his family, because his salary was too small, and what made the matter worse, was too irregularly paid to enable him to procure sufficient food for his table to afford him strength enough to perform the duties of his office, for he never would buy anything unless he paid for it at the time. His family were repeatedly whole days without any kind of meat, and would frequently sit down to the breakfast table with nothing before them but boiled corn and salt; no butter, no sugar, no bread, no beverage but cold water.

The clergy of this country, whether considered orthodox or not—for they all preach against vice and idleness and crime, and seek to promote industry, integrity, temperance, and Christian living—have a right to demand of the people a comfortable support for themselves and families, as much so as legislators and congressmen, for without the influences of the Christian religion, which clergymen maintain in existence by their labors, this government would become an anarchy, and murder and bloodshed would run rampant everywhere; for, aside from the restraining influence of the Christian system, no republican, no democratic government will ever succeed.

A magazine comes to us monthly, called the *Record*, a kind of missionary history. We always turn first to that portion of it which reports how much has been given the preceding month for the support of disabled and needy clergymen, or their destitute widows and penniless children; and it is an unfailing regret to notice that it is not ten times larger than it is. It is to be hoped that the time is not far distant when "societies" of every creed will consider it as much a disgrace not to provide handsomely for these classes, as it would be to turn a horse or dog, or servant out to die after a lifetime spent in arduous, faithful service.

A nation would shudder to hear that such men as Tyng, and Hall, and Cuyler, and Beecher, and Chapin, and Adams, and Bellows, and Burchard, and W. A. Scott, and others, after having given all their energies to church work for a quarter of a century and longer, should be compelled, in their old age, to go about and hunt up day's work, to keep them from starving. The churches would rally to their rescue, and untold thousands would be poured into the treasury for their relief. But there are many preachers, old and poor, and without a home, whose names are not known to fame, who are just as worthy of the public care; for, although their talents and capabilities are vastly inferior, they gave what they had; they gave their all to the church, and, whether that all was little or much, the principle of their consecration is the same, and they have a right to demand to be cared for; because it is a right, and not a charity; it is the man who "did it unto the least of these," who is pointed upwards, to follow the pathway to the skies. The world over, the man who dishonors an ambassador, dishonors the court which sent him. But what has this to do with a Journal of Health? Much every way! for, wherever there is a thriving church and a faithful minister, there is a constant diminution of poverty, and crime, and degradation, and want, and disease, and an increase in the elevation of public sentiment in favor of industry, integrity, temperance, and thrift; and where these are, there is prosperity, cultivation, and refinement. Let moneyed men have an eye to these considerations, and let them close their lives with liberal bequests for the benefit of the "world's workers," inasmuch as the Master has said, "The laborer is worthy of his hire."

